# Supplementary material for: The effect of proactive, interactive, two-way texting on 12-month retention in antiretroviral therapy: findings from a quasi-experimental study in Lilongwe, Malawi
Source: medRxiv. 2024 Jan 30:2024.01.26.24301855. Preprint. [Version 1] doi: 10.1101/2024.01.26.24301855 (PMC10863037; doi:10.1101/2024.01.26.24301855)
Supplement: Supplement 1 [file NIHPP2024.01.26.24301855v1-supplement-1.pdf]

Supplemental information

S1 File. Dataset. Retention outcomes dataset in CSV format.
